# G protein-coupled receptor 35: an emerging target in inflammatory and cardiovascular disease

**DOI:** 10.3389/fphar.2015.00041

**Published:** 2015-03-10

**Authors:** Nina Divorty, Amanda E. Mackenzie, Stuart A. Nicklin, Graeme Milligan

**Affiliations:** ^1^Molecular Pharmacology Group, Institute of Molecular, Cell, and Systems Biology, College of Medical, Veterinary and Life Sciences, University of Glasgow, GlasgowUK; ^2^Institute of Cardiovascular and Medical Sciences, College of Medical, Veterinary and Life Sciences, University of Glasgow, GlasgowUK

**Keywords:** GPR35, orphan receptor, zaprinast, kynurenic acid, CXCL17, heart failure, hypoxia, inflammation

## Abstract

G protein-coupled receptor 35 (GPR35) is an orphan receptor, discovered in 1998, that has garnered interest as a potential therapeutic target through its association with a range of diseases. However, a lack of pharmacological tools and the absence of convincingly defined endogenous ligands have hampered the understanding of function necessary to exploit it therapeutically. Although several endogenous molecules can activate GPR35 none has yet been confirmed as the key endogenous ligand due to reasons that include lack of biological specificity, non-physiologically relevant potency and species ortholog selectivity. Recent advances have identified several highly potent synthetic agonists and antagonists, as well as agonists with equivalent potency at rodent and human orthologs, which will be useful as tool compounds. Homology modeling and mutagenesis studies have provided insight into the mode of ligand binding and possible reasons for the species selectivity of some ligands. Advances have also been made in determining the role of the receptor in disease. In the past, genome-wide association studies have associated GPR35 with diseases such as inflammatory bowel disease, type 2 diabetes, and coronary artery disease. More recent functional studies have implicated it in processes as diverse as heart failure and hypoxia, inflammation, pain transduction and synaptic transmission. In this review, we summarize the progress made in understanding the molecular pharmacology, downstream signaling and physiological function of GPR35, and discuss its emerging potential applications as a therapeutic target.

G protein-coupled receptors are historically the most successful group of drug targets, and account for some 30–40% of approved drugs on the market today (Drews, 2000; Hopkins and Groom, 2002; Rask-Andersen et al., 2011). Indeed, several GPCR ligands are among the worldwide 100 best-selling pharmaceutical compounds (Zambrowicz and Sands, 2003). The success of GPCRs as therapeutic targets stems from their role as transducers of extracellular signals across the cell membrane and modulators of intracellular signaling, therefore bypassing the need for membrane-permeable ligands. Many GPCRs also contribute directly to the pathophysiology of disease, making them ideal as drug targets. However, despite their success, it is estimated that only 59 of the 370 non-olfactory GPCRs have been successfully exploited as drug targets, suggesting that the remainder may play largely modulatory roles unsuitable for this purpose or that there still exists great potential within the GPCR drug development arena (Sams-Dodd, 2005; Gashaw et al., 2011; Garland, 2013).

Orphan GPCRs (receptors that have not yet been associated with being activated by an endogenously produced ligand) provide a means by which to expand the repertoire of drug targets. For example, the orexin receptors, deorphanized in 1988, are the targets of the recently approved sleep-aid medication, suvorexant (Jacobson et al., 2014). Moreover, many other orphan or recently deorphanized GPCRs display therapeutically relevant tissue distribution profiles and/or have links to disease (Civelli et al., 2013; Garland, 2013). One such orphan GPCR is G protein-coupled receptor 35 (GPR35). However, despite this, in the 16 years since its discovery (O’Dowd et al., 1998), GPR35 remains poorly characterized and has been slow to amass interest. This stems from a lack of selective and potent tool compounds with which to probe GPR35 pharmacology and pathophysiology, with added complications including significant species selectivity issues (Jenkins et al., 2011, 2012; Funke et al., 2013; Neetoo-Isseljee et al., 2013) and the lack of consensus on the identity of the endogenous ligand (Mackenzie et al., 2011; Milligan, 2011). Here we provide an overview of updates in the field of GPR35 pharmacology, physiology and pathophysiology, and discuss how GPR35 is emerging as a GPCR with considerable therapeutic potential.

## ENDOGENOUS LIGANDS FOR GPR35

A number of endogenous molecules have been reported to act as agonists at GPR35 (**Table 1**). However, a number of these show modest potency at GPR35 and questions have been raised over whether they are likely to be produced at concentrations required for activation of GPR35. Moreover, some of the suggested ligands are certainly agonists at other GPCRs and/or pharma targets. An example of the latter, is LPA (mono-acylglycerol-3-phosphate). Derivatives of LPA, including 2-oleoyl LPA and 2-linoleoyl LPA, were shown to stimulate [Ca^2+^]_i_ mobilization above the level of [Ca^2+^]_i_ produced by endogenously expressed LPA receptors in HEK 293 cells stably expressing human GPR35 (Oka et al., 2010). Since a number of other ligands that activate GPR35 have not been shown to promote coupling to the Gα_q_ pathway (Jenkins et al., 2011, 2012; Mackenzie et al., 2011), this study could suggest a novel route for GPR35 stimulated signaling in which certain ligands are ‘biased’ in their function (Shonberg et al., 2014). However, the capacity of these [Ca^2+^]_i_ responses to be blocked by a GPR35 antagonist were not assessed, since no such ligands were available at that time and, therefore, this reported effect has not been intrinsically linked with GPR35 activity. Since LPA species certainly exert effects through a number of cellular proteins that form the specific sub-family of GPCRs named LPA_1-6_, it is unlikely that LPA acts with a potency that is selective toward GPR35 (Oka et al., 2010). Furthermore, LPA apparently does not stimulate recruitment of β-arrestin-2 to GPR35 (Southern et al., 2013), indicating that it acts in a manner distinct from the majority of other GPR35 ligands and, therefore, once again may display ‘bias’. Since LPA is associated with numerous pathological conditions including atherosclerosis (Schober and Siess, 2012), cancer (Gotoh et al., 2012), obesity, impaired glucose homeostasis (Rancoule et al., 2014), and pain (Ueda et al., 2013), there is a need to clarify the relevance of this reported effect of LPA at GPR35.

**Table 1:** Reported endogenous ligands with activity at GPR35.

Name	Structure	Action	Potency (EC_50_)	Comments	Reference
LPA	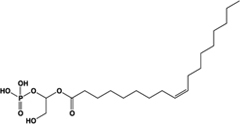	Unknown	Not reported	Phospholipid derivative; elevates [Ca^2+^]i through an unknown mechanism; activity at GPR35 not confirmed with a GPR35 antagonist; does not recruit β-arrestin-2	Oka et al. (2010), Southern et al. (2013)

Kynurenic acid	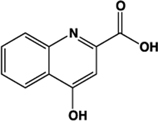	Partial agonist (Human) full agonist (Rat)	Human:** 217 μM** Rat:** 66 μM**	Metabolite of L-tryptophan	Wang et al. (2006), Jenkins et al. (2011)

cGMP	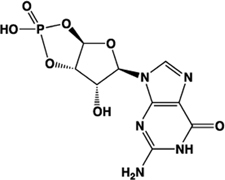	Partial agonist	Human: **131 μM**	Cyclic nucleotide derived from GTP	Southern et al. (2013)

DHICA	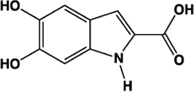	Partial agonist	Human:** 22 μM** (DMR 24 μM)	Intermediate in the biosynthesis of melanin	Deng et al. (2012a)

Reverse T3	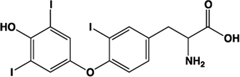	Full agonist	Human: **100 μM** (DMR 6 μM)	Hormone produced in the thyroid gland, liberated from precursor T4	Deng et al. (2012a)
CXCL17	Chemokine	Unknown	Not assessed	Elevates [Ca^2+^]i in a pertussis toxin sensitive manner	Maravillas-Montero et al. (2014)

Potency values in bold are those generated using a β-arrestin-2 recruitment assay. Action describes the efficacy of each ligand compared to zaprinast, if known.

The L-kynurenine metabolite kynurenic acid (4-oxo-1H-quinoline-2-carboxylic acid) is certainly able to promote activation of GPR35 (Wang et al., 2006). Although best known as a neuroprotective modulator through interaction with various receptors in the central nervous system (Mackenzie et al., 2011), many of these studies have not considered a role for GPR35, because early distribution studies suggested that this receptor is not highly expressed in the brain (O’Dowd et al., 1998; Wang et al., 2006). However, GPR35 has received attention as a potential modulator of the effects of kynurenic acid in the periphery (Moroni et al., 2012; Thorburn et al., 2014), although the level of kynurenic acid reported to occur in the gut and the concentration required to activate GPR35 does not appear to overlap, at least in humans (Jenkins et al., 2011; Milligan, 2011). In pharmacological assays, addition of kynurenic acid was found to mobilize [Ca^2+^]_i_ following transfection of GPR35 alongside a cocktail of Gα_q_-based G protein chimeras with a EC_50_ of 39 μM at human, 11 μM at mouse, and 7 μM at rat (Wang et al., 2006). Thus, a degree of species selectivity was observed between the human and rodent orthologs of GPR35 with kynurenic acid displaying higher potency at rat than at human (Wang et al., 2006). This profile was also observed in an independent study using HEK293 cells transiently expressing C-terminally manipulated forms of GPR35 and β-arrestin-2, which used a BRET assay, and generated an EC_50_ of 66 μM for rat GPR35-eYFP and >100 μM at the equivalent human construct (Jenkins et al., 2011).

In a concerted effort to deorphanize a panel of 82 orphan GPCRs, scientists from Medical Research Council Technology and GlaxoSmithKline screened 10,500 candidate endogenous ligands using the DiscoveRx PathHunter^TM^ β-arrestin-2 recruitment assay (Southern et al., 2013). In this screen, the second messenger cGMP was identified as a putative endogenous ligand of human GPR35. Subsequently, the derivatives MANT cGMP (2′-*O*-(*N*-methylanthraniloyl)-cGMP) and db-cGMP (dibutyryl-cGMP) were also identified as activators of human GPR35 (Southern et al., 2013). MANT-cGMP (EC_50_ 2 μM) was the most potent of these, with cGMP being the least potent (EC_50_ 130 μM; Southern et al., 2013). The functional relevance of these ligands at GPR35 remains to be demonstrated, including whether concentrations of cGMP present *in vivo* would be sufficient to activate GPR35. Notwithstanding, it is interesting to note that cGMP and its associated signaling pathways have received considerable attention recently for the treatment of cardiovascular disorders including hypertrophy, hypertension, ischemia, and reperfusion injury (Garcia-Dorado et al., 2009; van Heerebeek et al., 2012; Greene et al., 2013; Lukowski et al., 2014), and the possible link between GPR35 and cGMP may therefore deserve further attention.

An assessment of tyrosine pathway metabolic intermediates that contain catechol or carboxylic acid groups revealed that the melanin biosynthesis intermediate DHICA (5,6-dihydroxyindole-2-carboxylic acid) and thyroid hormone synthesis intermediates T3 (3,5,3′ triiodothyronine) and reverse T3 (3,3′,5′-triiodothyronine) promoted DMR in the human colorectal adenocarcinoma cell line HT-29 (Deng et al., 2012a). Application of the synthetic GPR35 antagonist CID-2745687 (methyl-5-[(tert-butylcarbamothioylhydrazinylidene)methyl]-1-(2,4-difluorophenyl)pyrazole-4 carboxylate) at a fixed concentration of 64 μM reduced the wavelength shift of each of the aforementioned agonists to almost basal levels, indicating that their activity occurred primarily through GPR35, which is expressed endogenously in these cells. However, confirmation of these responses in an independent assay utilizing a β-lactamase reporter gene-based readout of β-arrestin recruitment suggested that only DHICA acted with similar potency to that generated in the DMR format (Deng et al., 2012a). This indicated that T3 and reverse T3, if acting through GPR35, do so in a functionally selective manner. Reverse T3 was found to be the most potent of the tyrosine metabolites assessed, generating DMR in HT-29 cells with an EC_50_ of 5.9 ± 0.4 μM (Deng et al., 2012a). The EC_50_ observed in the Tango^TM^β-arrestin recruitment assay, however, was substantially higher, at 108 ± 9 μM. T3 was less potent than reverse T3, with an EC_50_ of 50 ± 5 μM in the DMR assay and 513 ± 61 μM in the Tango^TM^assay (Deng et al., 2012a). However, estimates of the free and total levels of T3 and reverse T3 in the literature (Chopra et al., 1975; Hüfner and Grussendorf, 1978; Prescott et al., 1979) indicate that it is unlikely that the concentrations of these molecules produced in man could activate GPR35 significantly *in vivo* (Deng et al., 2012a). Nonetheless, it is interesting to note that the levels of reverse T3 were found to increase, and T3 to decrease, following acute myocardial infarction (Friberg et al., 2002) and in advanced heart failure (Hamilton et al., 1990), and have been utilized as a measure to predict survival rates in patients with heart disease (Iervasi et al., 2003). Thus, akin to kynurenic acid, it appears that the levels of these molecules present in the plasma of humans are below the threshold required to activate GPR35 under normal physiological conditions, although their actions at GPR35 may be relevant when plasma levels are altered, for example during disease.

The most recent suggestion of an endogenous ligand for GPR35 is perhaps the most intriguing. Maravillas-Montero et al. (2014) have shown that the chemokine CXCL17 is able to elevate [Ca^2+^]_i_ in both HEK293 cells transfected to express human GPR35 and in the human monocytic cell line THP-1. Moreover, effects were produced at modest concentrations of the chemokine and this ligand was able to promote chemotaxis of THP-1 cells (Maravillas-Montero et al., 2014). The authors further highlighted similarities in sequence between GPR35 and more traditional chemokine receptors, going so far as to suggest the renaming of GPR35 as ‘CXCR8’ (Maravillas-Montero et al., 2014).

## SYNTHETIC LIGANDS FOR GPR35

Consensus may not have been reached on whether GPR35 is truly activated by any of the aforementioned endogenous ligands *in vivo* (Mackenzie et al., 2011; Milligan, 2011), yet progress in the identification of synthetic surrogate ligands for this receptor has been quite successful (**Table 2**). Focused compound discovery efforts carried out by both the industrial and academic sectors have reported a number of compounds that display moderate-to-high potency at GPR35, and indeed there now exists a reasonable number of tool compounds with which to probe the basic physiology and pathophysiology of this receptor. Notably, however, as observed for kynurenic acid, many of these synthetic compounds display marked species selectivity. For this reason, care must be taken when selecting the compound of choice for any form of study in rodents, because the majority of screens have been conducted initially on human GPR35 and markedly human-selective molecules will generate negative results in rodents.

**Table 2:** Potent synthetic ligands with activity at GPR35.

Name	Structure	Action	Potency (EC_50_/IC_50_)	Comments	Reference
Zaprinast, M&B 22,948	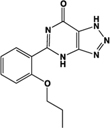	Full agonist	Human:** 2–8 μM** Mouse:** 1 μM** Rat:** 100 nM**	Xanthine derivative; cGMP PDE 5, 6, 9 inhibitor, selective for PDE 5 (IC_50_ 750 nM)	Taniguchi et al. (2006), Jenkins et al. (2012)

Pamoic acid	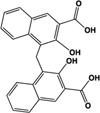	Partial agonist at human, weak activity at rodent	Human:** 30–50 nM** Mouse:** Inactive** Rat: **>100 μM**	Added as an “inert” substance to pharmaceutical agents; inhibits DNA pol β (IC_50_ 9 μM)	Jenkins et al. (2010, 2012), Neubig (2010), Zhao et al. (2010)

“Compound 1”	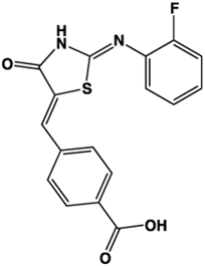	Full agonist at human, partial agonist at rodent	Human:** 26 nM** Mouse:** 17 μM** Rat:** 8 μM**	Novel ligand	Neetoo-Isseljee et al. (2013)

PSB-13253	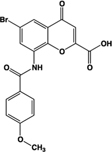	Full agonist at human, partial agonist at rat	Human:** 12 nM** Mouse: **Inactive** Rat:** 1.4 μM**	Novel ligand; [^3^H] radiolabelled structure also available with K_D_ of 5.3 nM	Funke et al. (2013), Thimm et al. (2013)

Lodoxamide, ICI 74917	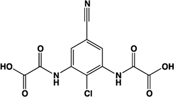	Full agonist	Human:** 4 nM** Rat:** 13 nM**	Mast cell stabilizer; ophthalmic solution: Alamide®; lodoxamide tromethamine, U-42,585E	Mackenzie et al. (2014)

Bufrolin, BRL10833	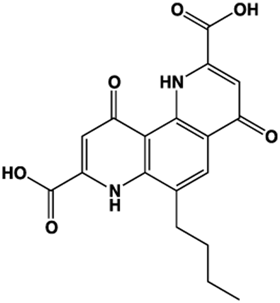	Full agonist	Human:** 13 nM** Rat:** 10 nM**	Mast cell stabilizer; equipotent at human and rat GPR35	Mackenzie et al. (2014)

ML145, CID-2286812	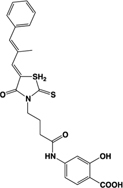	Inverse agonist	Human:** ∼25 nM**	Novel ligand; displays marked selectivity toward human GPR35	Heynen-Genel et al. (2010), Jenkins et al. (2012)

CID-2745687, SPB05142, ML194	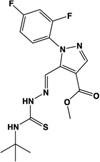	Inverse agonist	Human: ∼**200 nM**	Novel ligand; displays marked selectivity toward human GPR35	Zhao et al. (2010), Heynen-Genel et al. (2011), Jenkins et al. (2012)

Potency values in bold are those generated using a β-arrestin-2 recruitment assay. Action describes the efficacy of each ligand compared to zaprinast, if known.

One of the earliest and most useful ligands reported to have activity at GPR35 is the cGMP phosphodiesterase inhibitor, zaprinast (2-(2-propyloxyphenyl)-8-azapurin-6-one) (Taniguchi et al., 2006). Zaprinast tends to be employed as a reference agonist for GPR35 since it displays moderate-to-high potency at each of the human, mouse, and rat species orthologs (Taniguchi et al., 2006; Yang et al., 2010; Jenkins et al., 2012), and zaprinast’s GPR35-mediated effects can be dissected from those exerted through its inhibition of cGMP phosphodiesterases (Yoon et al., 2005; Taniguchi et al., 2006; Choi et al., 2007). Moreover, the capacity of a cGMP phosphodiesterase inhibitor to interact with GPR35 was, in part, the rationale for exploring whether cGMP might be an endogenous agonist of GPR35 (Southern et al., 2013). However, despite the wide application of zaprinast as reference agonist of GPR35, with reported potency of between 2 and 8 μM at the human ortholog, concerted efforts have been taken to identify agonists with high potency at human GPR35, and ideally one that acts with similar potency at the human and rodent orthologs.

A number of ligands that acted with potencies higher than zaprinast at the human ortholog were subsequently identified. These include pamoic acid (5-nitro-2-(3-phenylproplyamino) benzoic acid), which was identified independently by two groups following screens of the Prestwick Chemical Library®; (Jenkins et al., 2010; Zhao et al., 2010). However, the activity of pamoic acid at the rat and mouse orthologs, at least in the assays most often employed to screen for ligands at GPR35, is considerably lower than at human (**Table 1**), which prevents the use of this ligand in rodent studies (Jenkins et al., 2010, 2012; Milligan, 2011). This pattern of species selectivity was also displayed by “compound 1” (4-{(Z)-[(2Z)-2-(2-fluorobenzylidene)-4-oxo-1,3-thiazolidin-5-ylidene]methyl}benzoic acid) (Neetoo-Isseljee et al., 2013), and PSB-13253 (6-bromo-8-(4-[(3)H]methoxybenzamido)-4-oxo-4H-chromene-2-carboxylic acid) (Funke et al., 2013), which both display potency similar to pamoic acid at human GPR35 and act with significantly lower potency and efficacy at the rodent orthologs (**Table 1**). Compound 1 was identified following a screen of the Medical Research Council Technology 100,000 small molecule compound collection using the DiscoveRx enzyme complementation β-arrestin-2 recruitment assay (Neetoo-Isseljee et al., 2013), as were four other, chemically distinct, GPR35 agonists that display various profiles of species selectivity (Neetoo-Isseljee et al., 2013), while PSB-13253 was derived from a medicinal chemistry program based on derivatives of chromen-4-one-2-carboxylic acid (Funke et al., 2013).

The study by Funke et al. (2013) also presented the first comprehensive assessment of the structure-activity relationship between various chemical structures at human, mouse, and rat orthologs of GPR35. Although noted previously for individual ligands (Neetoo-Isseljee et al., 2013), this study provided collective evidence that GPR35 ligands act with either human-selective, human-and-mouse-selective, human-and-rat-selective or rodent-selective properties, with no ligands found to act with high and equal potency at all three orthologs (Funke et al., 2013). However, shortly after this study was published, two mast cell stabilizers were found to activate human *and* rat GPR35 in a high and equipotent manner, becoming the first tool compounds reported that could feasibly be employed to translate the findings obtained for the human receptor *in vitro* into *in vivo* physiological studies in rat models of physiology and disease (Mackenzie et al., 2014). In the BRET-based β-arrestin-2 recruitment assay, lodoxamide (2,2′-[(2-chloro-5-cyano-1,3-phenylene)diimino]bis[2-oxoacetic acid]) and bufrolin (6-butyl-4,10-dioxo-1,7-dihydro-1,7-phenanthroline-2,8-dicarboxylic acid) activated GPR35 with respective EC50s of 3.6 nM and 12.5 nM at human and 12.5 nM and 10 nM at rat (Mackenzie et al., 2014). This study also identified a number of mast cell stabilizers that act with rat-selectivity at GPR35 orthologs, including amlexanox and pemirolast (**Table 3**).

**Table 3:** Selection of other synthetic GPR35 ligands.

Name	Structure	Action	Potency (EC_50_)	Comments	Reference
Amlexanox, Amoxanox, AA-673	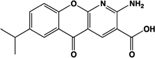	Partial agonist	Human:** 4 μM** Rat:** 23 nM**	Mast cell stabilizer; anti-asthma and anti-allergy medication; used to treat aphthous ulcers (Aphthasol®;); inhibits TBK1 and IKK-𝜀	Neetoo-Isseljee et al. (2013), Reilly et al. (2013), Southern et al. (2013), Mackenzie et al. (2014)

Cromolyn, sodium cromoglicate, DSCG	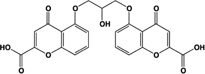	Full agonist	Human:** ∼7 μM** Mouse:** 22–56 μM** Rat:** ∼4 μM**	Mast cell stabilizer, prophylactic agent against asthma attacks (Intal®;); mast cell stabilizer and ophthalmic solution (Opticrom®;)	Yang et al. (2010), Jenkins et al. (2010), Deng et al. (2012a), Mackenzie et al. (2014)

Doxantrazole	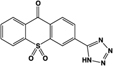	Partial agonist at human, full agonist at rat	Human:** 3.4 μM** Rat:** 300 nM**	Mast cell stabilizer	Mackenzie et al. (2014)

Furosemide	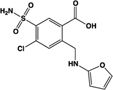	Partial agonist	Human: 8.3 μM ([Ca^2+^]i)	Loop diuretic; congestive heart failure and edema treatment; anti-asthma, anti-inflammatory agent	Yang et al. (2012), Neetoo-Isseljee et al. (2013)

Pemirolast, TBX	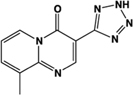	Full agonist	Human: **Inactive** Rat:** 95 nM**	Mast cell stabilizer, ophthalmic solution pemirolast potassium (Alamast®;)	Mackenzie et al. (2014)

Tyrphostin-51	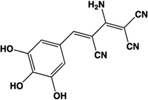	Full agonist	Human:** 8 μM** (DMR 120 nM)	Identified from a library of kinase inhibitors; prevents tyrosine phosphorylation	Deng et al. (2011a)

YE210	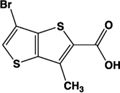	Full agonist	Human:** 15 μM** (DMR 64 nM)	Identified from a library of intermediates designed for non-linear optics and organic field-effect transistors	Deng et al. (2011b)

2,3,5-THB	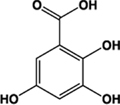	Partial agonist	Human:** 8.4 μM** (DMR 250 nM)	Metabolite of aspirin	Deng and Fang (2012)

Potency values in bold are those generated using a β-arrestin-2 recruitment assay. Action describes the efficacy of each ligand compared to zaprinast, if known.

## RECEPTOR HOMOLOGY MODELING EFFORTS INDICATE THAT THE BINDING POCKETS OF HUMAN AND RAT GPR35 ARE DISTINCT

Over the past 16 years, the lack of highly potent and species-equipotent tool compounds has greatly hindered progress in the elucidation of GPR35 function. However, assessment of the pharmacological differences between species has been useful in characterizing the ligand binding pocket of this GPCR. This approach was taken by Mackenzie et al. (2014), who used the differences in species selectivity at GPR35 to identify residues involved in ligand function. Based on the knowledge that the majority of GPR35 ligands contain at least one carboxyl group (Jenkins et al., 2010; Deng et al., 2011b, 2012b; Deng and Fang, 2012; Funke et al., 2013; Thimm et al., 2013), or a bioisostere of a carboxyl group (Mackenzie et al., 2014), it was postulated that positively charged residues within the generic ligand binding pocket of GPR35 that differed between human and rat might mediate the differences in potency. To this end, “species swap” mutations were carried out to switch key arginine residues positioned within proposed ligand binding regions (Venkatakrishnan et al., 2013) to the equivalent non-arginine residue in the opposite species ortholog. Mutagenesis efforts using these species swap mutations indicated that arginine residues present in extracellular loops 2 and 3 (or at the very top of transmembrane domain helices 6 and 7 (R164, R6.58, R7.32)) were important for ligand-induced β-arrestin-2 recruitment to human GPR35, while rat GPR35 was dependent on the species-conserved residue R4.60 at the extracellular end of transmembrane domain 4 (Mackenzie et al., 2014).

**FIGURE 1:**
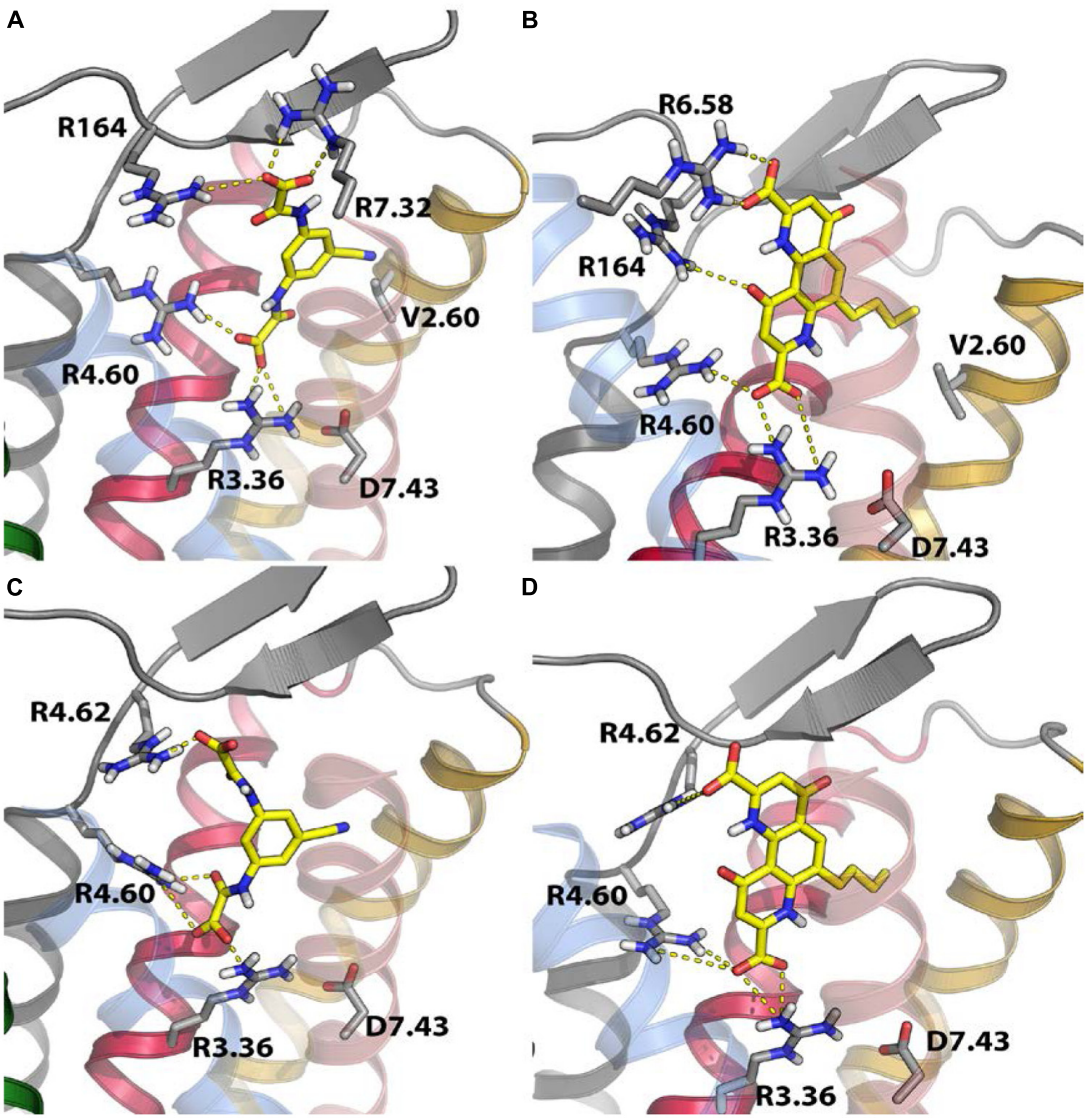
**Receptor homology modeling of human and rat GPR35 indicates residues important for ligand binding.** Based on homology modeling efforts with the protease-activated receptor 1 and mutagenesis studies, residues important in lodoxamide **(A,C)** or bufrolin **(B,D)** function at human **(A,B)** or rat GPR35 **(C,D)** were identified. The Figure is taken with permission, from Mackenzie et al. (2014).

Receptor homology modeling efforts based on the protease-activated receptor 1 structure (the most closely related GPCR to GPR35 for which an atomic level structure is known) indicated that the species-equipotent ligands behaved like human-selective ligands at the human receptor and rat-selective ligands at the rat receptor (Mackenzie et al., 2014), i.e., in order to generate an equipotent response the ligand must bind differently to each ortholog (**Figure 1**). A number of similar residues were implicated in GPR35 ligand binding in an independent study, in which alanine substitutions were generated at putative ligand-binding residues, and the resulting mutants functionally assessed in a number of distinct assay formats including β-arrestin-2 translocation, extracellular-signal regulated kinase phosphorylation and calcium mobilization (Zhao et al., 2014). In conjunction with receptor homology modeling efforts based on the β_2_-adrenoceptor, arginine residues at positions 4.60, 164, 167, and 6.58 were found to be involved in the binding and/or function of zaprinast and pamoic acid at human GPR35 (Zhao et al., 2014).

## GPR35 COUPLES TO BOTH Gα_**i**/**o**_ AND Gα_**13**_ SUBUNITS AND RECRUITS β-ARRESTIN-2

Alongside efforts to understand the mode of ligand binding to GPR35, downstream signaling and responses following GPR35 activation have been studied in an attempt to characterize pathways in which the receptor is involved and, thereby, understand the physiology and function of the receptor. Several signaling mediators that directly interact with GPR35 have been identified, and provide insight into the pathways and responses that could potentially be targeted through its pharmacological manipulation.

In terms of classical GPCR signaling, GPR35 appears to couple to various Gα subunits depending on the cell type and/or background species. Kynurenic acid-induced binding of [^35^S]-GTPγS induced by human GPR35 heterologously expressed in Chinese hamster ovary cells was inhibited by *Bordetella pertussis* toxin (Pertussis toxin), which is known to specifically disrupt coupling to Gα_i/o_ family G proteins (Wang et al., 2006). Furthermore, Ohshiro et al. (2008) demonstrated that in Chinese hamster ovary cells transfected with rat GPR35, kynurenic acid and zaprinast both inhibited forskolin-induced cyclic adenosine 3′ 5′ monophosphate (cAMP) generation, also indicating an involvement of one or more adenylate cyclase-inhibiting Gα_i/o_ family G proteins. GPR35-dependent effects on N-type calcium channels in rat neurons heterologously expressing human GPR35 have also been shown to be sensitive to Pertussis toxin (Guo et al., 2008). It is also interesting in this regard that the recent demonstration of CXCL17 as a potential GPR35 agonist indicated that both chemotaxis of HTP-1 cells and elevation of [Ca^2+^]_i_ levels in HEK293 cells transfected with the human ortholog of GPR35 were also blocked by pretreatment of the cells with Pertussis toxin (Maravillas-Montero et al., 2014).

As well as such effects, coupling to the Gα_13_ subunit, which activates guanine nucleotide exchange factors that promote GTP exchange to activate Rho family GTPases (Tanabe et al., 2004), has been observed in a human cell line. Zaprinast-induced activation of Gα_13_ was demonstrated using an activation state-sensing GTP-Gα_13_ antibody in transfected HEK293 cells (Jenkins et al., 2011). This was observed for human GPR35 and for rat GPR35. The same study also demonstrated the ability of zaprinast to induce [Ca^2+^]_i_ increases in HEK293 cells co-transfected with a chimeric Gα_q_/Gα_13_ G protein subunit and either human or rat GPR35, an effect that was specific for the Gα_13_ chimera compared with the related Gα_12_ subunit. However, no direct coupling to the G_q_/G_11_ group of G proteins, that are the routine transducers of phospholipase Cβ_1_-mediated elevation of [Ca^2+^]_i_ has been reported. The findings of specific roles for Gα_13_ are especially interesting considering that GPR55, the receptor phylogenetically closest to GPR35, also displays this unusual coupling specificity (Ryberg et al., 2007). Thus, the canonical G protein-mediated cellular and biological effects of GPR35 appear to be largely mediated by either Gα_i/o_ or Gα_13_ pathways. However, it is also possible that G protein coupling effectiveness might be dependent on additional factors that have yet to be explored systematically, such as ligand bias or Gα subunit expression. The possibility of ligand bias is particularly interesting in the context of GPR35 as a therapeutic target because, once deciphered, this feature could be exploited to increase the specificity of drug-induced responses. At present, current findings provide a basis for further study of these G protein-mediated pathways in the search for a function for GPR35.

As well as initiating G protein-dependent responses, GPR35 has been shown to directly recruit β-arrestin-2 upon stimulation with various agonists, including kynurenic acid and zaprinast (Jenkins et al., 2010). This is followed by agonist-dependent internalization of the receptor, which is presumably mediated by β-arrestin-2 as per the classical model for GPCR desensitization (Goodman et al., 1996; Mackenzie et al., 2014). For many GPCRs, β-arrestins have now also been shown to function as signaling scaffolds, interacting with a number of different pathways including the c-Jun N-terminal kinase, protein kinase B and cAMP pathways, but most notably the extracellular signal-regulated kinase (ERK1/2) pathway (Luttrell and Gesty-Palmer, 2010), in a G protein-independent manner. Whether this is the case for GPR35 remains to be determined, but given the diversity of processes seemingly influenced by GPR35, discussed below, screening for biased ligands may be key to understanding the physiological functions of this receptor and harnessing it as a therapeutic target.

## GPR35 AS A THERAPEUTIC TARGET

Amid uncertainty regarding the endogenous ligand of GPR35 and the complex nature of its G protein coupling profile, its physiological function and importance remain poorly understood. In recent years, however, the receptor has been associated with a range of diseases spanning multiple biological systems, which hints at its far-reaching potential as a therapeutic target. It will therefore be necessary to elucidate the physiological role of GPR35, both in health and disease, in order to utilize what we know of its pharmacology to develop it as a novel drug target.

### EXPRESSION PATTERN

*GPR35* mRNA transcripts have been detected in a number of diverse tissues throughout the body in both rodent and human studies. The publication that first identified GPR35 attempted to investigate tissue distribution through Northern blot analysis on various human and rat tissues (O’Dowd et al., 1998). Although no data were shown, the authors reported that mRNA expression was detected in rat intestine but that this was not replicated in human tissue (O’Dowd et al., 1998). Using the more sensitive approach of qRT-PCR, Wang et al. (2006) detected mRNA in lung, stomach, small intestine, colon, and spleen in both human and mouse tissues, and in human peripheral lymphocytes. *In situ* hybridization with *GPR35* RNA probes in a mouse tissue array revealed expression throughout the lower digestive tract, with hybridization in the duodenum, jejunum, ileum, cecum, colon, and rectum (Wang et al., 2006). Similarly, qRT-PCR in rat detected high levels of *GPR35* mRNA in lung, stomach, small intestine, and colon, but also skeletal muscle, dorsal root ganglion and uterus, with moderate expression in spinal cord, heart, liver, bladder, whole brain, and cerebrum (Taniguchi et al., 2006). Expression in the rat spinal cord and dorsal root ganglion has since been corroborated (Ohshiro et al., 2008; Cosi et al., 2011), as well as expression in mouse astrocytes (Berlinguer-Palmini et al., 2013). Finally, *GPR35* mRNA has also been found in various human immune cells, including mast cells, basophils, and eosinophils (Yang et al., 2010), and invariant natural killer-like T cells (Fallarini et al., 2010).

### GASTROINTESTINAL AND METABOLIC DISEASES

Based on the high expression levels of GPR35 in the gastrointestinal tract, it seems logical that the receptor should have a function in gut homeostasis. Indeed, GPR35 has been proposed as a risk factor for chronic inflammatory disorders of the gut such as IBD and ulcerative colitis. The first such report comprised a GWAS for early onset IBD (including Crohn’s disease and ulcerative colitis), which identified a *GPR35* SNP associated with ulcerative colitis (Imielinski et al., 2009). The SNP at rs4676410 is an upstream intron variant of GPR35 encoding a cytosine to thymine substitution, although no change in GPR35 expression was observed in tissue from individuals with IBD compared with that from genetically related controls. However, the same SNP was also associated with *CAPN10*, *KIF1A,* and *RNPEPL1* genes in a linkage disequilibrium block, and *CAPN10*, which encodes the calcium-regulated intracellular cysteine protease calpain 10, was found to be expressed at significantly lower levels in individuals with ulcerative colitis.

Prior to the IBD GWAS, the neighboring genes *CAPN10* and *GPR35* had both previously been linked with disease in a GWAS for type 2 diabetes mellitus (Horikawa et al., 2000). This study identified four non-synonymous SNPs in the coding region of *GPR35*, with only ‘UCSNP-38,’ which encodes a serine to arginine substitution at amino acid position 294, showing an association with type 2 diabetes. However, no evidence of linkage was found for *GPR35*, whereas ‘UCSNP-43’ in *CAPN10* displayed both association with type 2 diabetes and evidence for linkage. The study therefore concluded that the genetic variation in *CAPN10*, and not *GPR35*, was responsible for the disease susceptibility, and no further evidence for a role of GPR35 in type 2 diabetes has since been reported.

A more recent study into ulcerative colitis and primary sclerosing cholangitis, a chronic cholestatic liver disease, has further suggested involvement of GPR35 in IBD (Ellinghaus et al., 2013). The majority of primary sclerosing cholangitis patients have concurrent ulcerative colitis, and integrated analysis of GWAS studies for both diseases identified two GPR35 SNPs as having a significant association with primary sclerosing cholangitis (Ellinghaus et al., 2013). One of these was the rs4676410 upstream intron variant previously identified in the IBD GWAS (Imielinski et al., 2009), whereas the other, at rs3749171, encodes a methionine to threonine substitution in the third transmembrane domain of the receptor. The authors of this study speculated that, due to its expression pattern and the presence of kynurenic acid both in bile and in the gastrointestinal tract in disease (Forrest et al., 2003; Paluszkiewicz et al., 2009), GPR35 may have a role in the regulation of inflammation in the gastrointestinal and biliary tracts, although this is yet to be examined experimentally.

### CARDIOVASCULAR DISEASE

Although a role for GPR35 in cardiovascular disease is not immediately obvious from its expression profile, the receptor has been implicated, both directly and indirectly, in several aspects of cardiovascular dysfunction. The first study to suggest a cardiovascular role for GPR35 was a GWAS of hypertensive individuals, which aimed to identify novel predictors of coronary artery disease (Sun et al., 2008). This study identified an association between a non-synonymous *GPR35* SNP and coronary artery calcification, a risk factor in atherosclerosis and coronary artery disease. Interestingly, the identified rs3749172 SNP was the same one previously associated with type 2 diabetes (Horikawa et al., 2000). The SNP causes a serine to arginine substitution at amino acid position 294 in the receptor C-terminal tail, the primary domain involved in binding cytoplasmic proteins other than G proteins. The polymorphism may therefore influence β-arrestin-mediated responses and consequently alter the selectivity of the G protein- versus β-arrestin-dependent response. *In vitro* observations, however, do not support this hypothesis; the serine to arginine substitution was found not to alter the potency of a range of ligands in a PathHunter human GPR35-β-arrestin-2 interaction assay (Mackenzie et al., 2014). However, as serine 294 is a potential site of ligand-regulated phosphorylation, the importance of this SNP is certainly worthy of further investigation, not least because this variation is exceptionally common (reported minor allele frequency 0.494).

Further evidence of an involvement of GPR35 in cardiovascular disease was found in a global gene expression microarray analysis of twelve individuals diagnosed with chronic heart failure (Min et al., 2010). GPR35 expression in myocardial tissue from heart failure patients was significantly upregulated compared with that in healthy controls, and *GPR35* was among twelve genes selected for follow-up analysis based on its orphan status and its novelty as a potential therapeutic target. Adenovirus-mediated overexpression of GPR35 in primary rat cardiomyocytes resulted in reduced viability and cellular hypertrophy (Min et al., 2010). Measuring the hemodynamic parameters of *GPR35* knockout mice using a Millar catheter revealed a significant 37.5 mmHg increase in blood pressure in the knockout mice compared with wild type littermates, which strongly implicates GPR35 in blood pressure regulation (Min et al., 2010).

A more detailed investigation of GPR35 expression in neonatal mouse cardiomyocytes found both mRNA and cell surface protein levels to increase in response to hypoxia and hypoxia-inducible factor 1 activation (Ronkainen et al., 2014). Considering that hypoxia is a feature of most chronic cardiac pathologies, this provides a possible rationale for the apparently broad involvement of GPR35 in cardiovascular disease. In the same study, overexpression of GPR35 in neonatal mouse cardiomyocytes led to membrane ruﬄing and formation of retraction fibers, but no change in cell size. This conflicts with the previous finding by Min et al. (2010) in rat cardiomyocytes, suggesting possible species variation in the specific cellular effects of GPR35. Nonetheless, both results implicate GPR35 in regulation of actin cytoskeletal rearrangements. This is very likely to be mediated through the receptor’s coupling to Gα_13_ and its effector RhoA, which are well-established regulators of actin cytoskeletal remodeling (Siehler, 2009; Thumkeo et al., 2013).

In mouse surgical models of both acute hypoxia due to myocardial infarction and chronic pressure overload (a model of cardiac hypertrophy and heart failure), GPR35 expression is induced early on in the heart (after 1 day and 2 weeks, respectively; Ronkainen et al., 2014). In the chronic model this was found to precede pathological cardiac remodeling and the development of heart failure. These findings strongly suggest a role for GPR35 in cardiac pathophysiology, although further research is required to validate it as a therapeutic target in this context. Taken together, the human and mouse studies highlight potential uses for GPR35 as a predictive marker in the development of heart failure and as a target in the treatment of hypertension.

### IMMUNE HEALTH AND INFLAMMATION

GPR35 has been linked to inflammation, primarily in studies in which addition of GPR35 agonists has attenuated inflammatory processes, leading to the suggestion that GPR35 can modulate inflammatory conditions. GPR35 has been linked to immune health through dietary intake and digestion of tryptophan-containing foods such as red meat, fish, eggs, and vegetables, which are broken down to generate serotonin, melanin, kynurenic acid and nicotinamide, amongst others (Thorburn et al., 2014). In the gut, kynurenic acid can be liberated from dietary protein through the activity of *Escherichia coli* and transported to the extracellular milieu and blood where it elicits broadly anti-inflammatory effects (Kuc et al., 2008). Kynurenic acid was found to attenuate lipopolysaccharide-induced tumor necrosis factor-α secretion in human peripheral blood mononuclear cells and purified peripheral blood monocytes (Wang et al., 2006). In an independent study, kynurenic acid treatment of monocytes and neutrophils induced firm arrest of intracellular adhesion molecule 1-expressing monolayers of human umbilical vein endothelial cells (Barth et al., 2009). This signal was diminished by Pertussis toxin treatment and reduced by small hairpin-RNA delivery targeting GPR35, implicating GPR35 as a direct mediator of leukocyte adhesion (Barth et al., 2009). However, the potency of kynurenic acid in these studies was markedly higher than in any study reported to date that has employed transfection of the receptor into a heterologous cell background. In invariant natural killer-like T cells, which are involved in the maturation of dendritic cells and regulation of autoimmunity, kynurenic acid and zaprinast were shown to reduce interleukin-4 release into the culture medium in a dose-responsive and Pertussis toxin-sensitive manner without significantly reducing levels of interferon-γ, although the physiological significance of this is unclear (Fallarini et al., 2010).

In addition to studies based on kynurenic acid-induced effects, Yang et al. (2010) demonstrated GPR35 upregulation in response to challenge with IgE antibodies. The same study reported potency of anti-allergic, anti-asthma drugs cromolyn disodium and nedocromil sodium at GPR35, and an independent study also reported potency of several related mast cell stabilizers (**Tables 2** and **3**; Mackenzie et al., 2014). These studies suggest GPR35 may be involved in inflammation mediated by the innate immune response, and could potentially be targeted in the treatment of inflammatory conditions or autoimmunity, although a much more detailed understanding of the receptor’s role is required.

The recent report proposing GPR35 to be a chemokine receptor also suggests a role for GPR35 in the immune system (Maravillas-Montero et al., 2014). In this study, CXCL17 was shown to induce a [Ca^2+^]_i_ flux in a mouse pro-B cell line transfected with GPR35. CXCL17 is a macrophage chemoattractant, and is upregulated in idiopathic pulmonary fibrosis (Burkhardt et al., 2012). GPR35 expression, along with that of macrophage markers, was found to be significantly reduced in the lungs of *CXCL17* knockout mice, suggesting that GPR35-expressing macrophages require CXCL17 to migrate to the lungs, where they may play a role in inflammation (Maravillas-Montero et al., 2014). However, this is an isolated report that conflicts with some previous findings; therefore further investigation into this potential role for GPR35 is necessary.

### THE CENTRAL NERVOUS SYSTEM AND NOCICEPTION

G protein-coupled receptor 35 has also been suggested to have actions in the central nervous system following reports of its expression in rat spinal cord and dorsal root ganglion neurons (Ohshiro et al., 2008; Cosi et al., 2011; Moroni et al., 2012). Expression in dorsal root ganglion coincided with expression of transient receptor potential vanilloid subtype 1, a nociceptive neuronal marker (Ohshiro et al., 2008). Once again, kynurenic acid and zaprinast were shown to inhibit forskolin-induced cAMP production in a Pertussis toxin-sensitive manner in these neurons, implicating the GPR35/Gα_i/o_ axis as a potential target in the treatment of pain. In a mouse “writhing test” pain model, pre-treatment with the kynurenic acid precursor L-kynurenine or zaprinast significantly reduced the number of writhes by 58% and 54%, respectively (Cosi et al., 2011). GPR35, therefore, may be useful as an anti-nociceptive target, although whether this will translate into humans remains to be determined, especially since expression in human sensory neurons has not been reported to date.

A further link with the central nervous system is seen in mouse cortical astrocytes, in which *GPR35* mRNA was detected and kynurenic acid again inhibited forskolin-induced cAMP production, an effect that was abolished by either pre-treatment with the GPR35 antagonist CID-2745687 or *GPR35* mRNA silencing by siRNA (Berlinguer-Palmini et al., 2013). Once more, however, it must be noted that CID-2745687 has been reported to be highly selective for the human ortholog of GPR35 (Jenkins et al., 2012) bringing into question the mode of action of this compound in a mouse model. In the same study, both kynurenic acid and zaprinast altered [Ca^2+^]_i_ waves in these cells and reduced evoked excitatory post-synaptic currents in rat hippocampal neurons. Although kynurenic acid also acts on *N*-methyl-D-aspartate and α7 nicotinic receptors, the effects on intracellular calcium signaling and synaptic currents were insensitive to antagonists of these receptors, but sensitive to CID-2745687. In addition, the cGMP phosphodiesterase inhibitor sildenafil failed to reduce synaptic currents. This strongly suggests that the effects of kynurenic acid and zaprinast reported in this study are due to their agonism of GPR35 and not other known targets of these ligands. It is, therefore, possible that GPR35 plays a role in rodent synaptic transmission, although, once again, these results are yet to be tested in human cell models. Nevertheless, GPR35 remains a potential therapeutic target in the treatment of pain or the prevention of excitotoxic damage.

## CONCLUSION AND FUTURE PERSPECTIVES

The last few years have yielded significant advances in our understanding of GPR35 pharmacology and its physiological importance. Two major hurdles – a lack of pharmacological tools and significant species-selectivity – have been at least partially overcome, the former through the discovery and characterization of several novel synthetic ligands, and the latter through mutagenesis efforts that have provided insight into the cause of the species-selectivity, as well as the discovery of equipotent agonists. Future research efforts can now begin to assess the affinity of ligands for the receptor, which will be particularly useful in linking ligand-binding residues with functional capabilities. This has been made possible by the generation of the first radiolabeled ligand of GPR35: [^3^H]PSB-13253, which is an agonist with reported K_D_ of 5.3 nM at human GPR35 (Thimm et al., 2013). However, since this molecule is markedly human-selective, assessment of ligand affinity at the rodent receptors remains a challenge.

The increased repertoire of pharmacological tools can now be used to further probe GPR35 actions in physiological models. While current understanding makes GPR35 a promising candidate for therapeutic targeting given its involvement in a wide range of physiological and pathophysiological processes, no study has yet identified a definitive, targetable role for GPR35. This partially stems from a lack of tools; it is hoped that research efforts will now be directed toward the design of species equipotent antagonists, because the existing antagonists ML-145 (2-hydroxy-4-[4-(5Z)-5-[(E)-2-methyl-3-phenylprop-2-enylidene]-4-oxo-2-sulfanylidene-1,3-thiazolidin-3-yl ]butanoylamino]benzoic acid) and CID-275687 (Heynen-Genel et al., 2010, 2011; Zhao et al., 2010) act with almost complete human-selectivity, and are of no practical use in rodent models (Jenkins et al., 2012). Equipotent antagonists would enable definitive conclusions about the therapeutic value of GPR35 to be drawn by providing a means to probe off-target effects in rodent disease models. However, much of the work described here focuses on only rodent models, and so future research should also aim to translate these findings into clinically relevant models in order to truly assess the therapeutic value of targeting GPR35.

## Conflict of Interest Statement

The authors declare that the research was conducted in the absence of any commercial or financial relationships that could be construed as a potential conflict of interest.

## ABBREVIATIONS

BRET: Bioluminescence Resonance Energy Transfer
[Ca^2+^]_i_: intracellular Ca^2+^
cGMP: cyclic guanosine 3′ 5′ monophosphate
DMR: dynamic mass redistribution
EC_50_: half-maximal effective concentration
eYFP: enhanced yellow fluorescent protein
GPCR: G protein-coupled receptor
GWAS: genome-wide association study
HEK: human embryonic kidney
IBD: inflammatory bowel disease
LPA: lysophosphatidic acid
qRT-PCR: quantitative reverse transcriptase PCR
SNP: single nucleotide polymorphism.
